# An interactive image segmentation method for the anatomical structures of the main olfactory bulb with micro-level resolution

**DOI:** 10.3389/fninf.2023.1276891

**Published:** 2023-12-22

**Authors:** Xin Liu, Anan Li, Yue Luo, Shengda Bao, Tao Jiang, Xiangning Li, Jing Yuan, Zhao Feng

**Affiliations:** ^1^Britton Chance Center and MoE Key Laboratory for Biomedical Photonics, Wuhan National Laboratory for Optoelectronics, Huazhong University of Science and Technology, Wuhan, China; ^2^Research Unit of Multimodal Cross Scale Neural Signal Detection and Imaging, HUST-Suzhou Institute for Brainsmatics, Chinese Academy of Medical Sciences, Suzhou, China

**Keywords:** image segmentation, inception net, anatomical structure, three-dimensional reconstruction, main olfactory bulb

## Abstract

The main olfactory bulb is the key element of the olfactory pathway of rodents. To precisely dissect the neural pathway in the main olfactory bulb (MOB), it is necessary to construct the three-dimensional morphologies of the anatomical structures within it with micro-level resolution. However, the construction remains challenging due to the complicated shape of the anatomical structures in the main olfactory bulb and the high resolution of micro-optical images. To address these issues, we propose an interactive volume image segmentation method with micro-level resolution in the horizontal and axial direction. Firstly, we obtain the initial location of the anatomical structures by manual annotation and design a patch-based neural network to learn the complex texture feature of the anatomical structures. Then we randomly sample some patches to predict by the trained network and perform an annotation reconstruction based on intensity calculation to get the final location results of the anatomical structures. Our experiments were conducted using Nissl-stained brain images acquired by the Micro-optical sectioning tomography (MOST) system. Our method achieved a mean dice similarity coefficient (DSC) of 81.8% and obtain the best segmentation performance. At the same time, the experiment shows the three-dimensional morphology reconstruction results of the anatomical structures in the main olfactory bulb are smooth and consistent with their natural shapes, which addresses the possibility of constructing three-dimensional morphologies of the anatomical structures in the whole brain.

## 1 Introduction

The olfactory sensation is highly significant for rodents, which involves the recognition of predators and foods, the formation of memories, and the adjustment of reproducing and feeding behaviors (Ashwell, 2012). The olfactory system of rodents comprises several brain regions, in which the most rostrally located main olfactory bulb (MOB) plays an important role in connecting the olfactory epithelium of the nasal cavity, and downstream higher functional areas, such as olfactory tubercle, piriform area, and entorhinal cortex (Luo, 2015).

Within MOB, the olfactory pathway is topologically organized with single-cell resolution. The glomeruli, which are generally globular with 40–189 μm in diameter, and which are located in the glomerular layer of MOB (GL), are the key elements integrating and dispatching the olfactory information of the pathway (Royet et al., 1988; Ashwell, 2012). Every glomerulus gathers the 1 μm or thinner axons emitted from the olfactory receptor neurons (ORN) widely distributed on the olfactory epithelium that expresses the same receptor (Royet et al., 1988).

Therefore, to precisely dissect the olfactory pathway in MOB, the projection route and target of which should be located with single-cell resolution, thus urging to create a map that contains the precise three-dimensional morphologies of the anatomical structures in MOB with 10 to even 1 μm spatial resolution.

However, to the best of our knowledge, there are no studies of three-dimensional reconstruction with such high spatial resolution. This is because the shapes of anatomical structures in MOB are so complicated that the normal image acquisition techniques are not capable of completely resolving them. Giving the mitral layer of MOB (ML) as an example, this circular layer extends as broad as 140 μm range, while the narrowest part of which is only 5 μm wide, approximating to the diameter of single neuron soma (Quan et al., 2013). Nevertheless, the spatial resolution of MRI techniques is generally hundreds of microns per pixel, thus being unable to obtain the morphological details of ML; traditional optical imaging techniques can reach micron or even sub-micron resolution in horizontal direction though, the axial resolution of which is still limited to tens or hundreds of microns per level, resulting in providing insufficient information for reconstructing precise and intact volumes of these anatomical structures in MOB as well (Hess et al., 2018).

Recently developed micro-optical imaging techniques are capable of obtaining cytoarchitectural images with micron resolution in both horizontal and axial directions. However, technical challenges still exist in recognizing the boundaries of MOB anatomical structures from such images and reconstructing their three-dimensional morphologies (Li et al., 2010; Gong et al., 2013; Yuan et al., 2015). Due to the large size of micro-optical images and the complex features of anatomical structure, it is unrealistic to directly apply the successful segmentation methods in MRI to micro-optical images (Feng et al., 2016).

Given the gap, some researchers tried to use statistical-based segmentation to identify the distribution of the cells or other indicators to determine the boundaries of nuclei (Mesejo et al., 2013). However, the validity of those methods depends on the accuracy of the cell detection procedure, which itself is a challenging issue. There are also some works (Xu et al., 2020) on end-to-end segmentation by constructing a traditional segmentation model which is sensitive to parameter settings.

Similarly, some researchers also introduce deep learning to the segmentation framework of micro-optical images. Chen et al. (2019) trained a classifier to learn various cytoarchitectural properties, which was then combined with certain registration techniques to locate the nuclei in the brainstem. This method determines the initial location of the nuclei by a few manually labeled data, which is prone to manual error. Some researchers (Wang et al., 2021; Qu et al., 2022) build fully convolutional neural networks based on U-Net (Ronneberger et al., 2015) or DeepLabV3+ (Chen et al., 2018) to coarsely segment brain regions with large volumes. Zhang et al. (2019) used a one-shot learning strategy that took both the atlas and the images as the input of the fully convolutional neural network, which solved the problem of lack of labeled training data. Li et al. (2023) addressed a multiview semi-supervised segmentation network to accurately segment brain regions with several annotated brains. With the development of the vision image transformer (Dosovitskiy et al., 2020), some larger networks, like TransUNet (Chen et al., 2021) and Swin-Unet (Cao et al., 2021), were applied to medical images and addressed competitive performance. However, these methods take the downsampled images as the input of the fully convolutional neural network due to the limited computing power and acquire rough segmentation results with low resolution which lacks details and is inaccurate.

Here, we proposed an interactive volume image segmentation framework for segmenting anatomical structures with micro-level resolution in the horizontal and axial direction. The proposed method is constructed by combining manual annotation and automatic segmentation. The sparse manual annotation provides both the training data and the localization of anatomical structures to be segmented, while the neural network automatically segments the rest slices to construct a dense annotation. A patch strategy with an annotation reconstruction process is applied to learn the precise features of the images with fast inference time. The accuracy of our method was 81.8% evaluated by DSC, which was higher than other methods. The average time cost for segmenting multi-target areas on every coronal slice with 1 μm/pixel resolution was within 4 min, which was remarkably fast. In this article, we addressed a patch-based neural network and a novel post-processing strategy to recognize anatomical structures in micro-optical images with super-high resolution. To the best of our knowledge, we reconstructed the three-dimensional morphologies of the anatomical structures in the MOB of the mouse brain with both the axial and horizontal resolution of 1 μm/pixel for the first time. The method proposed in this article may provide insight into precise and fast segmentation of anatomical structures with micro-level resolution.

## 2 Materials and methods

### 2.1 Framework

**Figure 1:**
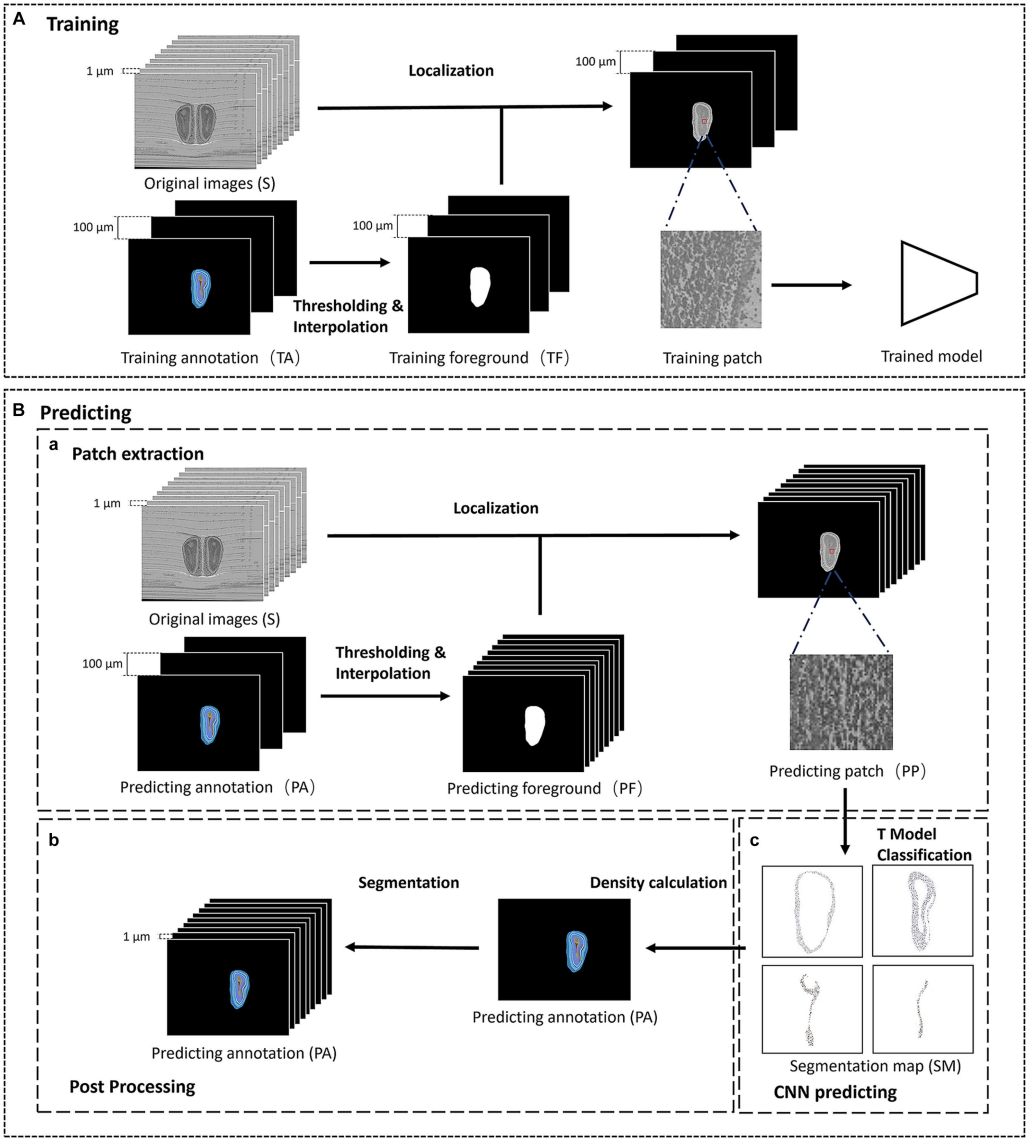
The framework of the segmentation method. **(A)** The training phase of the segmentation method. **(B)** The predicting phase of the segmentation method. **(a)** The patch extraction process of the predicting phase. **(b)** The post processing of the predicting phase. **(c)** The CNN predicting part of the predicting phase.

The proposed interactive segmentation framework can be divided into two parts: the training and predicting part, as shown in Figure 1. The training part contains initial localization and the training of the patch-based convolutional neural network (CNN). To obtain the training data and initial location of the anatomical structures, we first select some olfactory bulb slices at equal intervals on the original image as training slices, and manually annotate the anatomical structures in the training slices as training annotations. Then, we extract patches from the training slices and train a patch-based CNN to learn the precise local texture features of different structures. The predicting part mainly includes predicting data extraction, CNN prediction, and annotation reconstruction. Excluding the training slices, the remaining slices are used as predicting slices. Since the massive computation of classifying each pixel, we randomly extract patches from the predicting slices and send them to the trained model for prediction. Then we reconstruct the pixel-level predicting annotations after post-processing. Finally, the final segmentation results of anatomical structures are composed of the training annotations and the predicting annotations.

### 2.2 Initial localization

Note that it is challenging to locate the region of interest in the micro-optical images with high resolution, we manually annotate some olfactory bulb slices at intervals to initially locate the anatomical structures. Firstly, we select a olfactory bulb slice at interval *n* on the original image containing *c* (*c* ≥ 1) anatomical structures and obtain *p* (*p* < =100) olfactory bulb slices as training slices (TC, TC = {TC_1_, TC_2_, …, TC_p_}), while other olfactory bulb slices are used as the predicting slices (PC, PC = {PC_1_, PC_2_, …, PC_np-n_}). Then we annotate the anatomical structures in TC, and get the training annotation. Each anatomical structure in the training annotation is assigned a specific one-hot label.

Traversing training annotation, we separate the foreground area that contains all anatomical structures through binarization, and perform morphological expansion operation on the binarized foreground area, then we get the initial foreground area corresponding to TC_i_. After locating the anatomical structures in TC, we use the nearest neighbor method to interpolate *n* images between TC_i_ and TC_i + 1_ in turn, and the interpolated images are used as the initial foreground area of each olfactory bulb slice between PC_ni_ and PC_ni + n_. The initial location of the anatomical structures is composed of the initial foreground area in TC and PC.

### 2.3 Training of neural network

**Figure 2:**
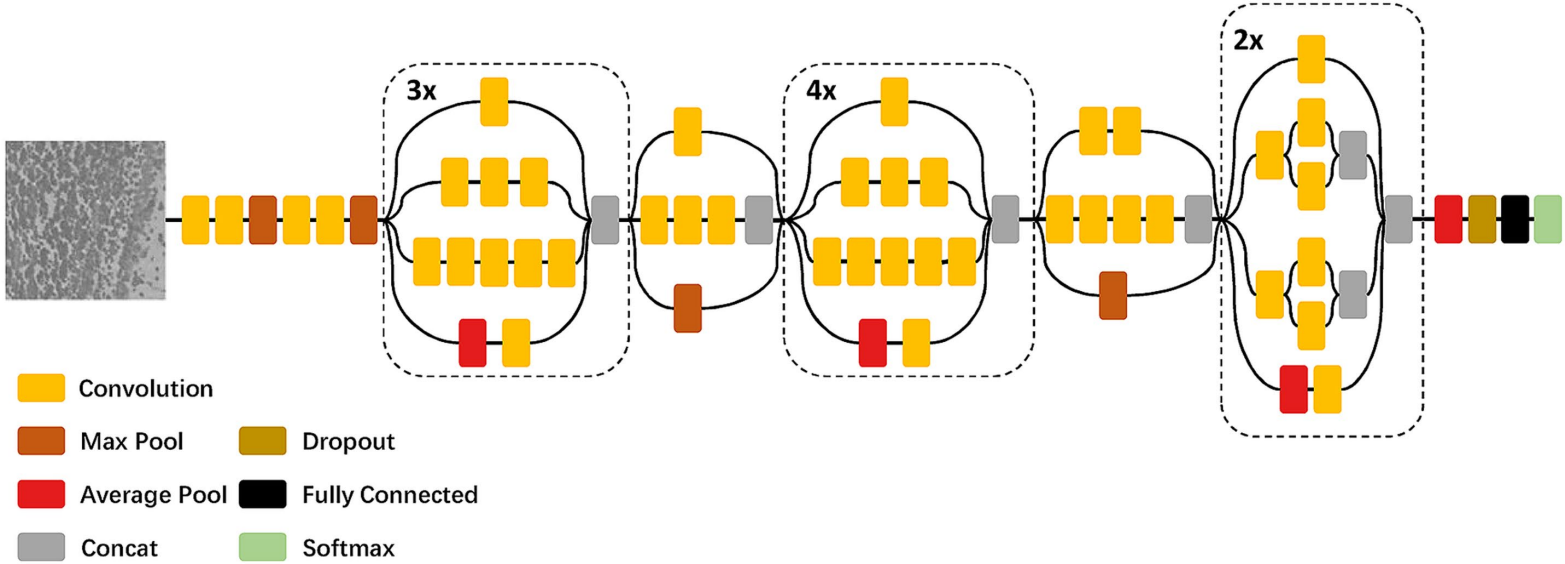
Architecture of the patch-based neural network modified by InceptionV3.

Compared to the fully convolutional neural network which focuses on the global information in images with low resolution, we build a patch-based neural network modified by InceptionV3 (Szegedy et al. 2016) to learn the texture information of each anatomical structure (Figure 2). The number of the output nodes in the last fully-connected layer is the same as the number of anatomical structures to be segmented, and the dropout (Hinton et al., 2012) is also added in front of the fully-connected layer to prevent overfitting. To reduce the amount of training parameters and the cost of training time, the input image of the InceptionV3 network is downsampled to a resolution of 100 × 100 pixel^2^ and normalized.

For the training data, we randomly select *m* patches with the size of *p* × *p* in the foreground area of each slice, and record the labels of the center points of these patches in the corresponding annotation. 70% of the *m* data are used as training patches, and 30% are used as validating patches. After training, we obtain the trained model which is capable of classifying 10 regions such as GL; the external plexiform layer of MOB (EPL); ML; the inner plexiform layer of MOB (IPL); the granule cell layer of MOB (GCL); the accessory olfactory bulb (AOB); the anterior olfactory nucleus (AON); the subependymal zone (SEZ); the anterior commissure, olfactory limb (aco); and the lateral olfactory tract, general (lotg).

### 2.4 Annotation reconstruction

In general, the network should predict every pixel in the slices to obtain the segmentation results, but the high resolution of the micro-optical images makes it time-consuming. Therefore, we randomly sample some patches to predict and reconstruct the predicting annotation. According to the initial location of the anatomical structures, we selected *m* patches with the size of *p* × *p* on PC_i_ as the predicting patches of PC_i_. Repeating the above operations on PC, we get the predicting patch set.

For PC_i_, we construct a corresponding sparse map (SM) with *c* channels, while each channel corresponds to the probability that each pixel on PC_i_ belongs to the corresponding anatomical structure of this channel. We traverse each patch in the predicting patch set and record the coordinates of its center point. Then we send each patch into the trained model and get a *c*-bit one-hot label. Each bit of the one-hot label corresponds to the probability that the center point of each patch belongs to a given anatomical structure. We fill the obtained *c*-bit one-hot codes into their corresponding pixels of the SM according to the recorded coordinates and finally obtain the SM with *c* channels which can be calculated by following formula:


(1) $$SM\left(x,y\right)=\mathrm{OneHot}\left(M\left({PC}_{i}\left(x,y,p\right)\right)\right)$$

where *x* and *y* are the coordinates of the center point in PC_i_, *p* is the patch size, M is the trained model, $\mathrm{OneHot}\left(•\right)$ converts the output of M to the *c*-bit one-hot label, and ${PC}_{i}\left(x,y,p\right)$ is the corresponding predicting patch.

Next, we calculated the signal density on the channel of SM corresponding to the anatomical structure *t*. The signal density calculation means using a 50 × 50 pixel^2^ window to scan each pixel. We calculate the average gray value of all pixels within the window, which illustrates the confidence that the center pixel belongs to the anatomical structure *t*. Repeat the above calculation to obtain the confidence map of each channel. Lastly, we compare the confidence of each pixel among different channels and determine the anatomical structure to which the pixel belongs by choosing the identifier number of the channel with the highest confidence value, thus obtaining a rough segmentation result (RR) which can be calculated by following formula:


(2) $$RR\left(x,y\right)=\mathrm{Softmax}\left(\overset{25}{\underset{i=-25}{\sum }}\overset{25}{\underset{j=-25}{\sum }}SM\left(x+i,y+j\right)\right)$$

where $\mathrm{Softmax}\left(•\right)$ calculates the class with the highest confidence.

Since some anatomical structures in the rough segmentation result consist of multiple small-area connected domains which are usually false positive results, we also perform post-processing to further optimize the rough segmentation result. The post-processing is to redistribute the connected domains of each anatomical structure in the rough segmentation result and appoint the false positive connected domains with smaller areas to their closest anatomical structure to obtain a more precise prediction annotation.

Finally, through iterative predicting of PC, the predicting annotations is obtained. Since the original image is composed of TC and PC, the segmentation result of the original image, is composed of the predicting annotations and the training annotations according to the order of the olfactory bulb slices in S.

### 2.5 Quantitative evaluation

To quantitatively evaluate the segmentation performance of our method, we apply the dice similarity coefficient (DSC), which is a popular metric for medical image segmentation evaluation to score the similarity between predicted segmentation and ground truth (Müller et al., 2022). Since the segmentation performance is related to the accuracy of the classifying network, precision, and recall are also added. Precision indicates the model’s capability to detect the background class in an image, while recall evaluates the capabilities for correctly identifying true negative classes (Müller et al., 2022). These metrics are calculated as follows:


(3) $$DSC\;\left(A,B\right)=\frac{2\times |A\cap B|}{\left|A|+|B\right|}$$

(4) $$\mathrm{Precision}\left(A,B\right)=\frac{\left|A\cap B\right|}{\left|A\right|}$$

(5) $$\mathrm{Recall}\left(A,B\right)=\frac{\left|A\cap B\right|}{\left|B\right|}$$

Here for each anatomical structure, A and B represent the binary images with automatically segmented results and ground truth respectively, |A| and |B| represent the total number of pixels with a value of 1 in the binary image, and |A∩B| represents the total number of pixels with a value of 1 in both A and B.

### 2.6 Data preparation

For the experiment data, we used the Nissl-stained brain images from C57BL/6 J adult mice acquired by the Micro-optical sectioning tomography (MOST) system (Li et al., 2010). The mice were 8-week-old males, and the original resolution of the obtained image dataset was 0.32 μm in the horizontal direction and 1 μm in the coronal and axial direction. We downsampled the dataset in the horizontal direction to a resolution of 1 μm (11,400 × 9,000 × 13,200 pixel^3^) to ensure the speed of segmentation.

### 2.7 Test environment

The experimental environment of this method consists of one GPU server, which is equipped with four NVIDIA V100 GPU cards, a 12-core CPU (Intel Xeon-6126w × 2), and 192 GB memory. The software resources of this method are listed in Table 1.

**Table 1:** Software resources.

Software	Link
PyTorch v.1.7.0	https://pytorch.org/
Keras v.2.6.0	https://keras.io/
PyCharm v.2021.2	https://www.jetbrains.com/
Anaconda v.5.2.0	https://www.anaconda.com/
Python v.3.6.5	https://www.python.org/

## 3 Results

### 3.1 Performance

To train the neural network, the batch size of our model was set as 100, the epoch was 50, the applied optimizer was Adam optimizer (Kingma and Ba, 2014), and the learning rate was 1e−4. Also, an early stopping strategy was adopted to prevent overfitting which would stop training if the validation loss did not decrease within five epochs. The learning rate planning strategy was applied so that the learning rate would be reduced by half for every five epochs.

To evaluate the performance of our method, we selected 10 anatomical structures in the olfactory area as the target to be segmented. Our data consisted of 13,200 coronal slices, 2,900 of which contained the olfactory areas. So we selected these 2,900 coronal slices as S in which 29 coronal slices were further selected at 100 μm intervals as TC, while the remaining ones formed PC. We got TA by manually annotating in TC and randomly selected 220,000 training patches as TP. We used TP to train the designed CNN and got a model T to segment PC. To evaluate the performance of our method, 14 coronal slices were selected as test slices. The ground truth of target anatomical structures in test slices was labeled by three experts.

**Figure 3:**
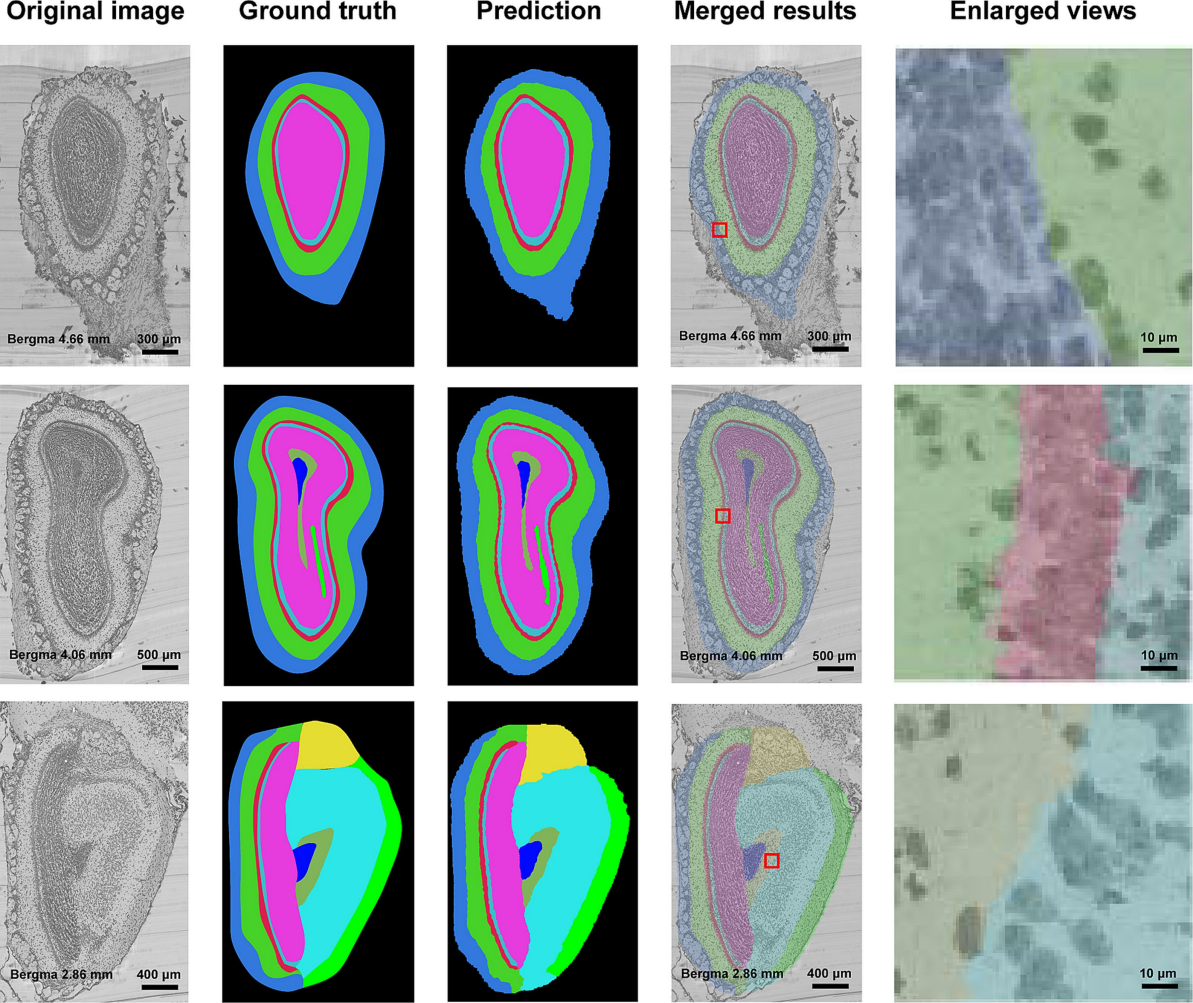
Segmentation results of anatomical structures in olfactory areas. The first column is the original image, the second column to the fourth column are the ground truths, the segmentation results, the superimposed image of the segmentation results and the original image, and the enlarged view of the red box in the superimposed image. Different colors represent different anatomical structures. Cornflower blue: GL, Lime green: EPL, Indian red: ML, Medium turquoise: IPL, Deep pink: GCL, Yellow: AOB, Cyan: AON, Blue: SEZ, Dark sea green: aco, and Green: lotg.

**Figure 4:**
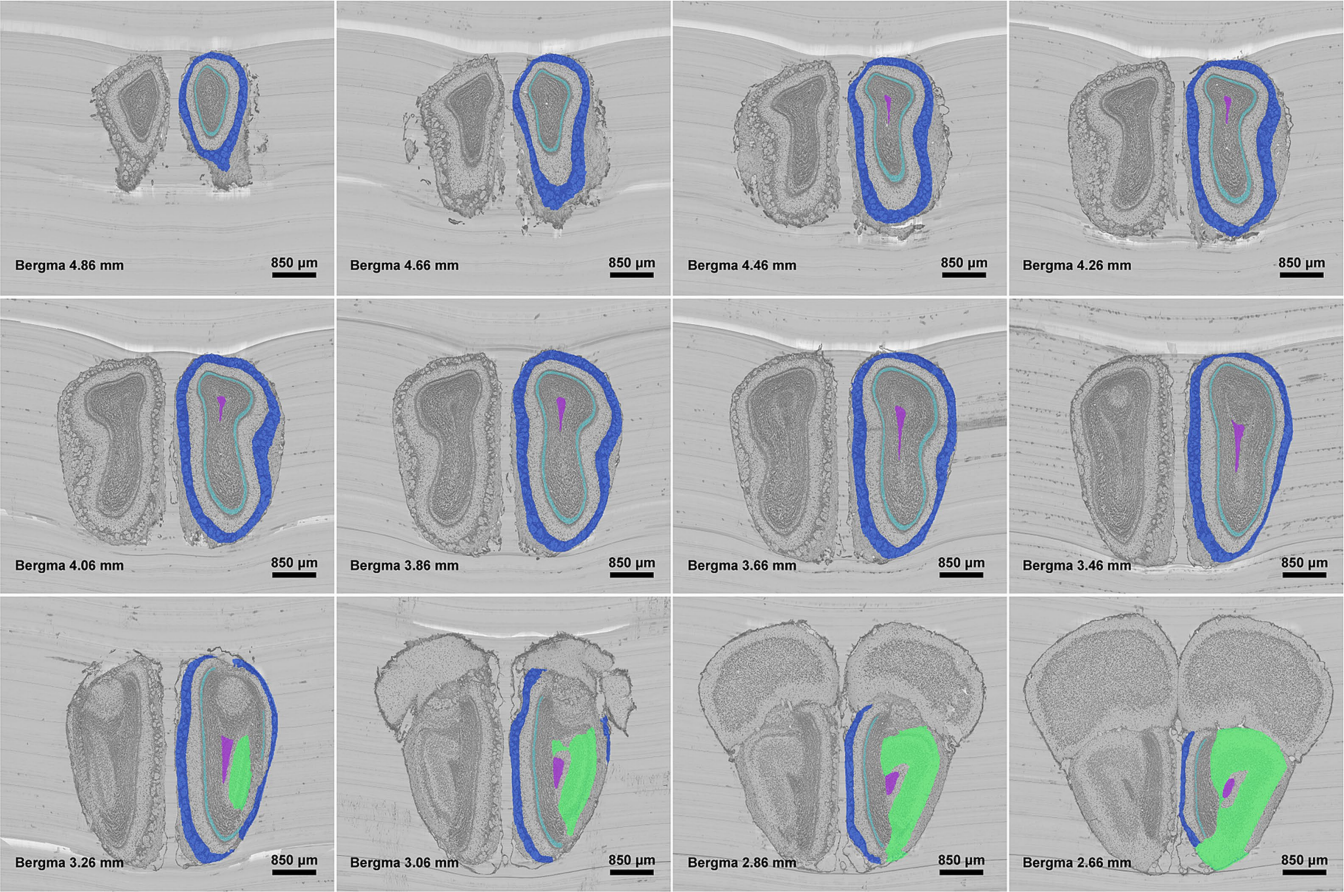
Localization results of different anatomical structures on different olfactory bulb slices. Different colors represent different anatomical structures. Royal blue: GL, Pale turquoise: IPL, Medium orchid: SEZ, and Spring green: AON.

Figure 3 shows the segmentation results of our method for all anatomical structures in the olfactory area. It can be seen from the figure that the real shape of each anatomical structure is completely preserved. Additionally, from the enlarged view, we can see that the segmented boundaries are very close to the natural boundaries between neighboring anatomical structures. Figure 4 shows the segmentation results of specific anatomical structures on consecutive coronal slices. The morphological changes of four anatomical structures along the axial direction can be seen from this figure, which also proves that our method can still obtain better segmentation results even if the morphology of the target structure varies sharply along coronal slices.

**Figure 5:**
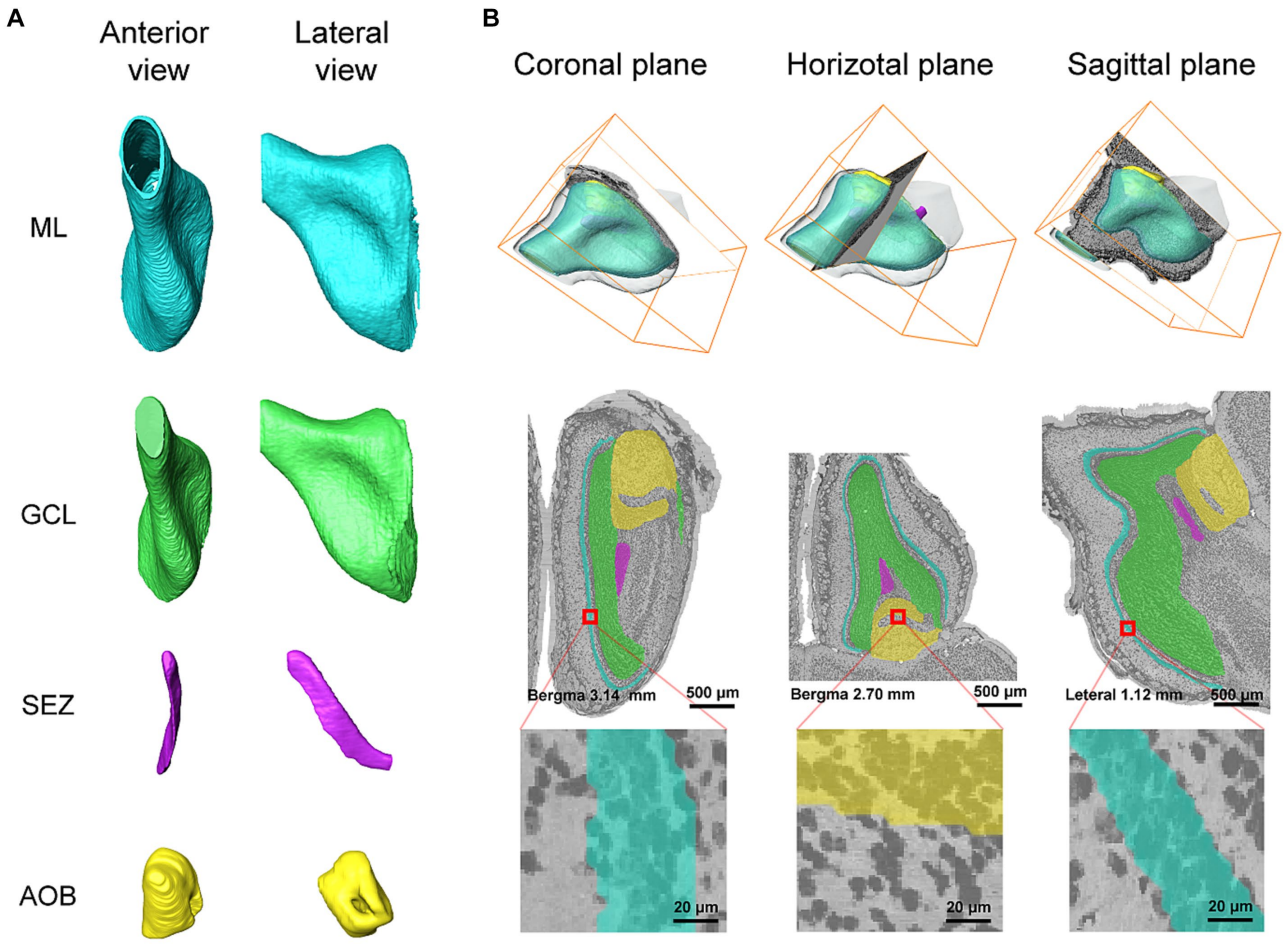
**(A)** The anterior and lateral view of 3D reconstruction results for ML, GCL, SEZ, and AOB. **(B)** The reslices of segmentation results in coronal, sagittal, and horizontal planes. Different colors represent different anatomical structures. Cyan: ML, Green: GCL, Medium orchid: SEZ, and Yellow: AOB.

Since our method segments the boundaries of anatomical structures on 2D images, while the anatomical structures are three-dimensional objects, it is necessary to verify the continuity of the segmented boundaries along the axial direction. Figure 5 shows the three-dimensional reconstruction of the target anatomical structures and their reslices on sagittal and horizontal planes. The reconstructed surfaces of the three anatomical structures are smooth, and are consistent with their natural shapes as well, which demonstrates the segmented contour of each anatomical structure by our method is also continuous in the axial direction.

**Figure 6:**
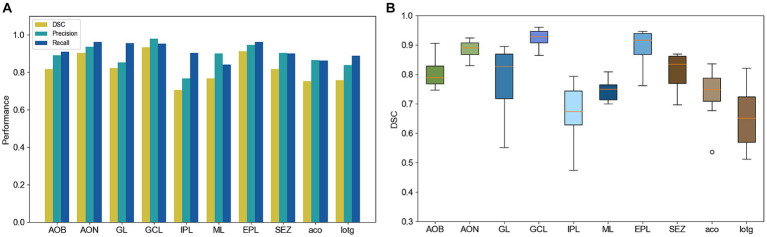
**(A)** Quantitative performance of different anatomical structures. **(B)** DSC box plot of different anatomical structures.

In addition, we quantitatively evaluated the segmentation results of the anatomical structures in the olfactory area on 14 test slices. We calculated the similarity between the segmentation results and the ground truth of the anatomical structures. Figure 6A shows the DSC, precision, and recall of the segmentation of each anatomical structure. The DSC of most anatomical structures exceeds 0.7, the precision and recall exceed 0.8, and these three indices of some anatomical structures such as GCL and EPL, even exceed 0.9. Figure 6B shows a box plot of the DSC for each anatomical structure. The average DSC of 10 anatomical structures is higher than 0.8. Even for IPL which has a slender and complex shape, the average DSC is also higher than 0.6.

### 3.2 Comparison with other methods

**Figure 7:**
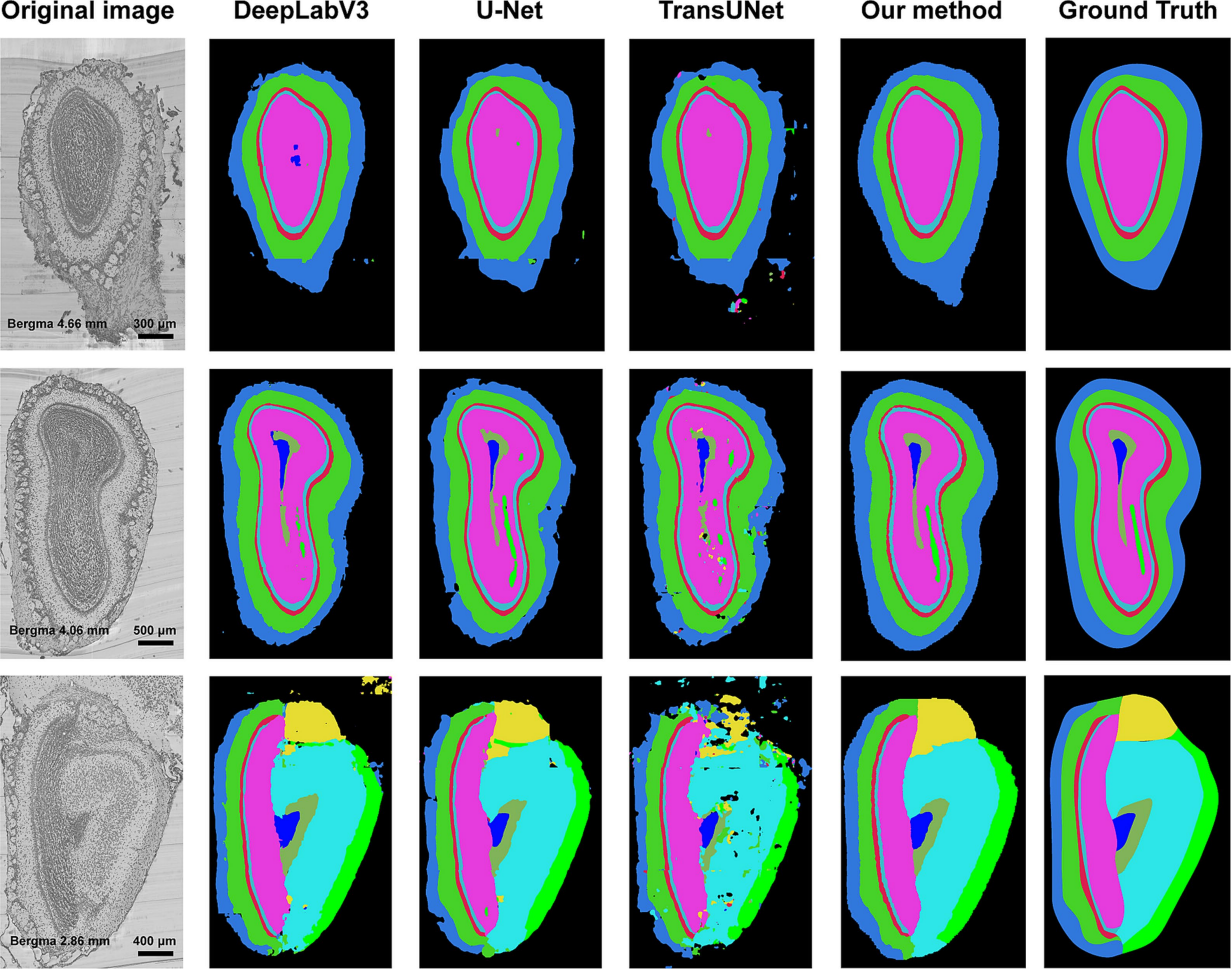
Segmentation results of different methods. The first column is the original image, and the second column to the fifth column is the segmentation results of DeepLabV3, U-Net, TransUNet, and our method. The last column is the ground truth of segmentation. Different colors represent different anatomical structures. Cornflower blue: GL, Lime green: EPL, Indian red: ML, Medium turquoise: IPL, Deep pink: GCL, Yellow: AOB, Cyan: AON, Blue: SEZ, Dark sea green: aco, and Green: lotg.

In this section, we compared our method with other segmentation methods, including DeepLabV3 (Chen et al., 2018), U-Net, and TransUNet. 29 coronal slices were used as TC and 14 coronal slices as test slices. Due to the large size of the slice, the input and output of DeepLabV3, U-Net, and TransUNet were both the sampled patches. Specifically, the input size of these three models were both 512 × 512 pixel^2^ constraint by the capacity of GPU memory. Figure 7 shows the segmented boundaries of target structures by all the methods. The first column is the original images, and the last five columns are the segmentation results of different methods and the ground truths. We can see that there are some problems in the segmentation results of the compared methods, such as irregular shapes, unsmooth boundaries, and wrong locations of anatomical structures. However, our method not only acquires accurate boundaries but also gets the most similar shapes and locations to the ground truths, which proves that our method can be well applied to segmentation task of the anatomical structures.

Furthermore, we quantitatively compare the segmentation results of different methods. Table 2 shows the DSC, precision, and recall of the four methods, each value shown in this table is the average of the performance on 10 anatomical structures. It can be concluded that our method obtains the highest scores.

**Table 2:** Performance evaluation of different methods.

Method	DSC	Precision	Recall
DeepLabV3	0.752 ± 0.081	0.848 ± 0.044	0.855 ± 0.075
U-Net	0.763 ± 0.083	0.878 ± 0.050	0.850 ± 0.070
TransUNet	0.672 ± 0.174	0.809 ± 0.079	0.768 ± 0.182
Our methods	**0.818 ± 0.072**	**0.886 ± 0.058**	**0.913 ± 0.041**

The bold values means the highest metrics of different methods.

### 3.3 Selection of receptive field and sampling number

**Figure 8:**
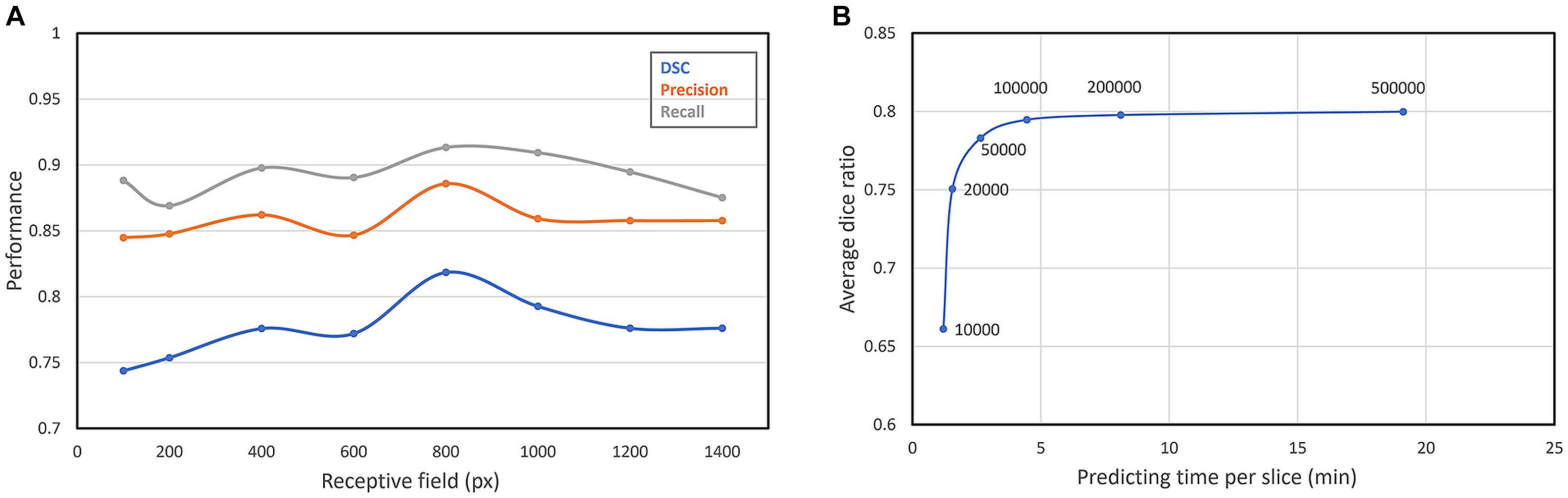
**(A)** Segmentation performance of different receptive fields; **(B)** DSC and predicting time of different patch number.

The receptive field is a crucial factor that can affect the segmentation performance. In the convolutional neural network, the size of the receptive field changes with the input size. The patch with a large receptive field contains not only the texture characteristics of the anatomical structures but also the relative position information among their neighboring anatomical structures. However, an excessively large receptive field will lead to a decrease in the discrepancy among adjacent patches, resulting in low-efficient recognition capacity and inaccurate segmentation boundaries. To find the best receptive field size, we consecutively set seven different receptive fields of our network. As can be seen from Figure 8A, with the increasing of the receptive field, the segmentation performance improves continuously, until the receptive field reaches the peak when the receptive field is 800 × 800 pixel^2^. When the receptive field continues to increase, the segmentation performance begins to decrease slowly, so we finally set the receptive field of our neural network to 800 × 800 pixel^2^.

The number of sampling patches is also an important parameter. Due to the high resolution of our original images, only part of all extracted patches will be selected for prediction. The more patches are, the more accurate the segmentation result will be, but also more time cost will be required. We select six different patch numbers for testing the best patch number. As can be seen from Figure 8B, with the increase of the patch number, the segmentation performance also improves. However, when the average DSC increases to about 0.8, it converges gradually to a constant, but the time cost for prediction still increases linearly. Therefore, we choose 100,000 as the best patch number which decreases the predicting time per slice in 4 min.

## 4 Discussion

In this paper, we propose an interactive volume image segmentation framework for the anatomical structures with micro-level resolution in the horizontal and axial direction. Firstly, we get the manual annotation with interval, and then use the patch-based convolution neural network to learn the texture features of each anatomical structure, and to classify the pixels in the image. Finally, the precise reconstruction is performed by the density calculation and post-processing. Our method can segment multiple anatomical structures simultaneously through one network, and the segmentation results have high accuracy while the morphology of the anatomical structures can be preserved. Compared with DeepLabV3, U-Net, and TransUNet, our method can achieve higher accuracy.

We showed the two-dimensional segmentation results and three-dimensional reconstruction results of the anatomical structures in MOB, which were the same as the natural morphology of the anatomical structures. Even if the interval between the manually annotated slices was large, the segmentation results obtained still had good continuity in the axial direction, which was very suitable for the accurate localization of the anatomical structures. The accuracy of our method is not affected by individual variations due to the combination of human involvement. Since the reliable results on MOB in this article, we believe that our method has the potential to be the basis of a user-interactive annotating tool, assisting in illustrating the brain atlas and other anatomical structures annotating tasks in the future.

Nevertheless, there still needs further improvement in our approach. Our method needs manual annotation, which is difficult for people without anatomy knowledge. Also, although the existing methods can segment a coronal slice in 4 min, due to the large size of the whole brain image set, a faster method is still needed for large-scale processing in the future. Therefore, our future work is to build an automatic and faster segmentation framework of anatomical structures.

## Data availability statement

The original contributions presented in the study are included in the article/supplementary material, further inquiries can be directed to the corresponding author.

## Ethics statement

The animal study was approved by Animal Ethics Committee of the Huazhong University of Science and Technology. The study was conducted in accordance with the local legislation and institutional requirements.

## Author contributions

XLiu: Conceptualization, Data curation, Formal analysis, Methodology, Software, Validation, Visualization, Writing – original draft, Writing – review & editing. AL: Data curation, Funding acquisition, Project administration, Resources, Supervision, Writing – review & editing. YL: Conceptualization, Data curation, Writing – review & editing. SB: Data curation, Visualization, Writing – review & editing. TJ: Data curation, Resources, Writing – review & editing. XLi: Resources, Writing – review & editing. JY: Data curation, Resources, Writing – review & editing. ZF: Conceptualization, Funding acquisition, Methodology, Project administration, Resources, Writing – review & editing.
